# Automatic Detection of the Running Surface of Railway Tracks Based on Laser Profilometer Data and Supervised Machine Learning

**DOI:** 10.3390/s24082638

**Published:** 2024-04-20

**Authors:** Florian Mauz, Remo Wigger, Alexandru-Elisiu Gota, Michal Kuffa

**Affiliations:** Institute for Machine Tools and Manufacturing, ETH Zürich, 8092 Zurich, Switzerland

**Keywords:** railways, rail profiles, condition monitoring, laser profilometer, surface condition, running surface

## Abstract

The measurement of the longitudinal rail profile is relevant to the condition monitoring of the rail infrastructure. The running surface is recognizable as a shiny metallic area on top of the rail head. The detection of the running surface is crucial for vehicle-based rail profile measurements, as well as for defect detection. This paper presents a methodology for the automatic detection of the running surface based on a laser profilometer. The detection of the running surface is performed based on the light reflected from the rail surface. Three rail surfaces with different surface conditions are considered. Supervised machine learning is applied to classify individual surface elements as part of the running surface. Detection by a linear support vector machine is performed with accuracy of >90%. The lateral position of the running surface and its width are calculated. The average deviation from the labeled widths varies between −1.2 mm and 5.6 mm. The proposed measurement approach could be installed on a train for the future onboard monitoring of the rail network.

## 1. Introduction

The share of public transportation in Switzerland is to be increased in the future, as described by Nold et al. [1]. This has led to an increased demand for rail-based transport and is leading to the greater exploitation of the rail infrastructure. To ensure reliable and safe operation, the maintenance of the infrastructure is essential. Tanaka et al. [2] describe a preventive maintenance concept using rail grinding to minimize the occurrence of rail corrugation. Zoeteman et al. [3] show the implementation of the preventive maintenance concept in the Netherlands. Dhillon [4] identifies inspection as one of seven elementary components of a preventive maintenance strategy. This means that the current condition of the rail surface must be monitored regularly. The running surface is of particular interest as the wheel–rail contact occurs in this area of the rail surface. Edel et al. [5] described the emergence of a lowered and broadened running surface due to the formation of squats. Satoh et al. [6] investigated the crystal orientation of the material in the area of the running surface as a result of the damage caused by operation. Wang et al. [7] investigated the detection of rail wear in the area of the running surface based on acquired images. The running surface is also relevant to the generation of rolling noise. Thompson [8] showed that the roughness of the contact surfaces of the rail and the wheel correlates with the rolling noise. Acoustic rail grinding can improve the acoustic properties, as described by Kuffa et al. [9]. Rail roughness can be measured to determine the condition of the running surface. According to Cordier et al. [10], a distinction can be made between direct and indirect measurement methods. Direct measurement methods are often tactile methods that measure the longitudinal profile at a defined lateral position on the rail surface. Alternatively, trolley devices are applied, such as the corrugation analysis trolley (CAT) developed by Grassie et al. [11]. The longitudinal rail profile must be measured following EN 13231-2 [12] in the area between the highest point of the rail surface and up to 15 mm towards the inner edge of the rail. Chen et al. [13] have developed a positioning system for a measuring device installed on the vehicle to measure the longitudinal rail profile. The system aims to measure at the center of the rail head. The running surface is not detected and consequently not taken into account. EN15610 [14] recommends that the position of the running surface is defined manually before measuring the acoustic rail roughness. Mauz et al. [15] have presented a vehicle-based system for the measurement of rail roughness. Information on the lateral position of the running surface is necessary for the effective application of the system, as the vehicle can introduce a lateral offset relative to the rail surface. Both cameras and laser sensors are suitable for detecting the running surface. Camera images are dependent on different lighting conditions. Laser profilometers have previously been applied to detect the condition of the rail surface based on distance measurements. Ye et al. [16] presented a methodology for the detection of rail surface defects. Mauz et al. [17] showed that an artificial running surface in a laboratory environment could be detected using a laser profilometer. The intensity of the light reflected from the surface was determined to distinguish between different surface conditions. Compared to a camera, the profilometer has the advantage of not being dependent on the lighting conditions. In contrast to a single-point laser sensor, a laser scanner measuring on a line has the benefit of detecting the running surface of the rail over a certain width. This approach can be applied to the various rail surface conditions that exist in the rail network.

**Figure 1:**
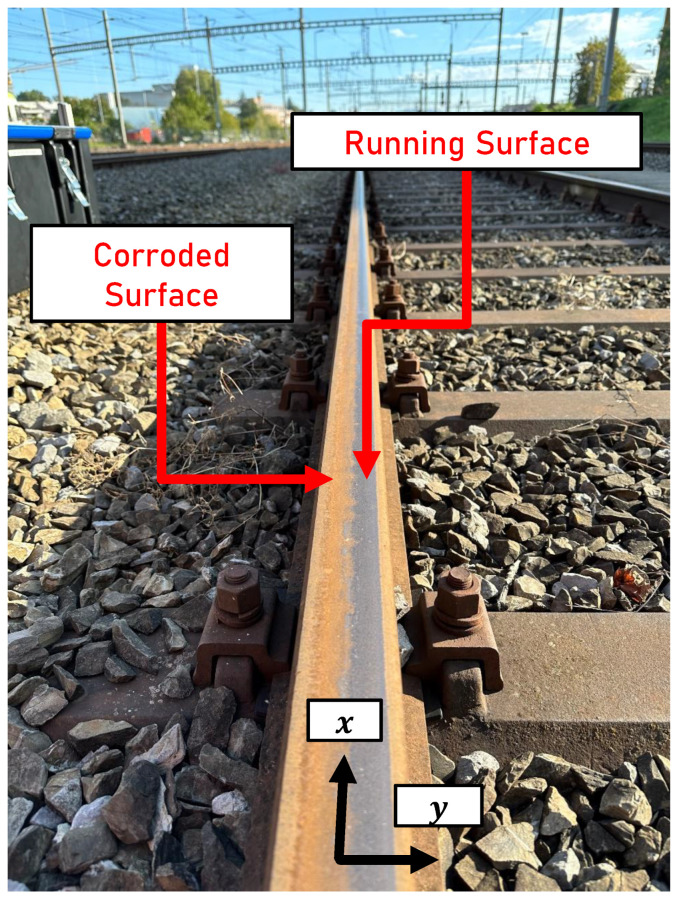
Reference system of the rail and indication of the corroded areas of the rail surface and the running surface. *x*: longitudinal rail direction, *y*: lateral rail direction.

In the following, the running surface or running band is defined as the non-corroded and shiny part of the rail surface, where the contact between the wheel and rail takes place. Figure 1 illustrates a rail surface with an established running surface.

The areas of the rail surface not traversed by the wheel are corroded or dirty and provide a contrast to the reflective surface of the running surface. The width and lateral position of a distinct running surface are the result of the sum of all wheel–rail contacts within the respective track section. Different widths $w_{s}$, lateral positions $y_{s}$ and conditions of the running surface can be encountered on the surface of the rail in the rail network.

This paper uses field measurements during running surface detection to validate the results of a study in the laboratory. Three different rail surfaces with different characteristics of running surfaces are considered. A methodical procedure for the detection of the running surface based on supervised machine learning is proposed. Supervised machine learning is applied to ensure that detection is automated and can be used for a wide variety of different rail surface conditions. The aim is to monitor the width $w_{s}$ and lateral position $y_{s}$ of the running surface in the reference system of the rail.

## 2. Materials and Methods

### 2.1. Experimental Setup

**Figure 2:**
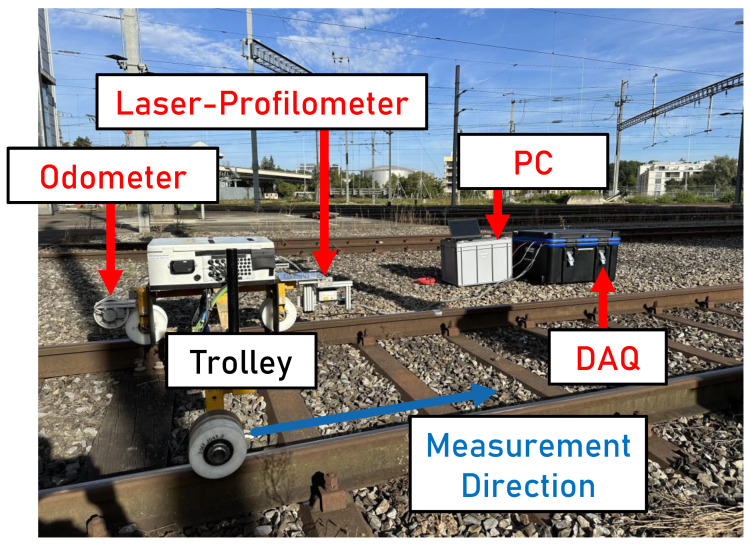
The experimental setup on the track and the data acquisition (DAQ) setup next to the track.

The measuring setup comprises a trolley device that can be moved manually. The trolley setup, including the equipment for data acquisition during the measurement, is shown in Figure 2.

**Figure 3:**
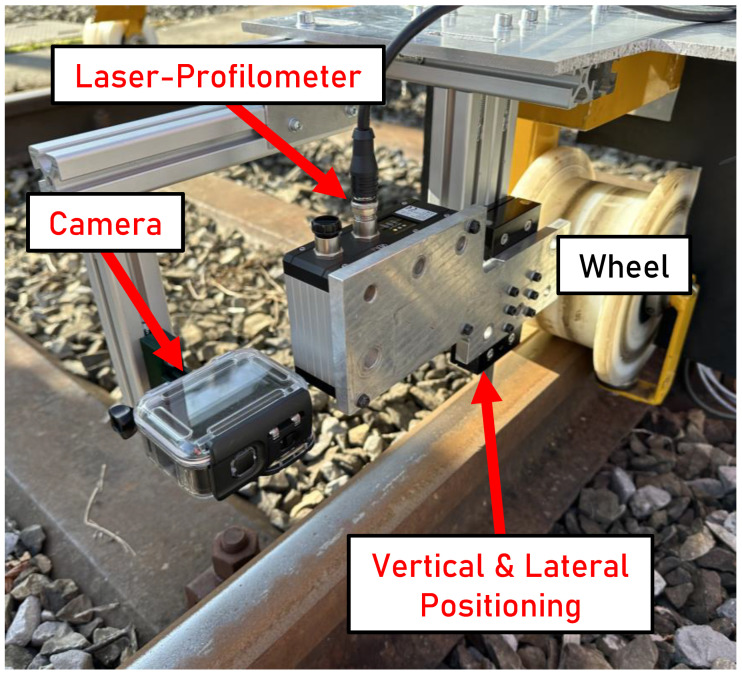
Installation of the laser profilometer with the vertical and lateral positioning mechanisms and the camera on the trolley device.

A scanCONTROL 3060-50/BL laser profilometer from Micro-Epsilon is mounted on the trolley using a vertical positioning mechanism at a distance of 125 mm from the rail surface. The lateral mounting position of the laser profilometer above the rail surface can be offset perpendicularly to the direction of travel. The attachment of the laser profilometer to the trolley is shown in Figure 3.

In addition to the laser profilometer, a camera used for the labeling of the data is mounted on the trolley device. The laser profilometer projects a line perpendicular to the direction of travel with a length of approximately 51 mm. The distance to the rail surface and, simultaneously, the intensity of the light reflected by the surface is measured at a sampling frequency of 30 Hz. The intensity is categorized into 1024 increments. No reflected light hitting the receiving element of the profilometer corresponds to an intensity of 0 and the complete reflection of the emitted light equates to an intensity of 1023. The laser profilometer is connected to a computer by a network card. The longitudinal position is detected by a Hengstler RI58-O encoder connected to a measurement wheel. The encoder signal used for the odometer is recorded with an NI9402 module, a cRIO 9045 controller, and the LabVIEW 2022 Q3 software from National Instruments. The sampling frequency of the encoder is set to 30 kHz. An average longitudinal sampling distance dx of 4.06 mm is obtained. On average, 1988 data points are received in the lateral direction. The average lateral sampling distance dy amounts to 0.025 mm.

The following lateral positions of the laser profilometer relative to the rail head are considered.

- **Central:** The lateral position of the laser profilometer is centered towards the middle of the railhead.
- **Left:** From the central lateral position, the laser profilometer is shifted 10 mm to the left.
- **Right:** From the central lateral position, the laser profilometer is shifted 10 mm to the right.

**Figure 4:**
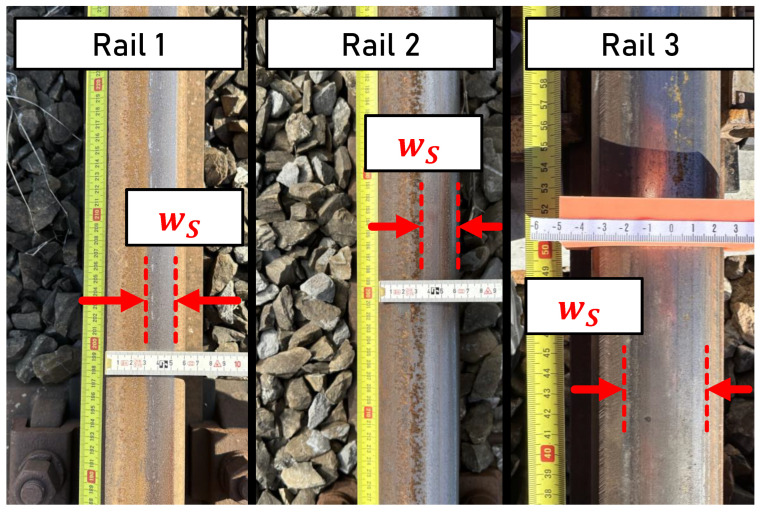
Surface conditions of the measured rails: Rails 1 and 2—close to a depot and hence not often frequented; Rail 3—regularly frequented.

Three rails with different surface conditions are considered. On each rail, a section of 5 m is marked with aluminum tape that is attached to the rail surface. Measurements are conducted for each rail and are repeated three times. Figure 4 shows the rail surfaces of the individual rails measured.

The rails represent different surface states, which allow the consideration of varying running surface patterns.

- **Rail 1** is located in front of a depot for rail vehicles and is rarely frequented. Slight corrosion within the running surface is evident.
- **Rail 2** is also located in front of a depot for rail vehicles. Compared to Rail 1, Rail 2 is more frequently operated due to the regular operation of vehicles.
- **Rail 3** is located on a regularly frequented section of the track. At the time of measurement, the section of road was closed for 3 to 4 days due to construction works. The rail surface is characterized by a wide and established running surface.

### 2.2. Detection Approach

**Figure 5:**
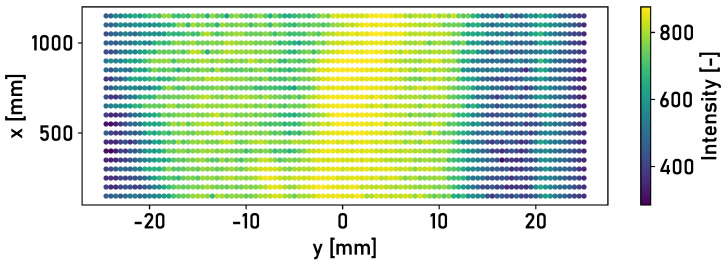
The detected intensity of the surface of Rail 1, divided into surface elements with an element size of 0.5 mm × 50 mm (Δy × Δx). The elements are colored according to the average intensity of the respective element.

The automated detection of the running surface must be effective for all rail surfaces. The application of fixed threshold values on single data points is therefore not suitable for the detection of the running surface. Features are calculated for surface elements defined on the rail surface. The classification of the surface elements as “running surface” or “no running surface” is based on supervised machine learning. Random forest, k-neighbors and linear support vector machine models are applied to the classification problem. These are particularly suitable for classification problems for which only a limited quantity of data is available. Figure 5 shows the rail surface, divided into elements that are colored according to their average intensity.

The element size of 0.5 mm × 50 mm (Δy × Δx) is an empirical compromise between the filter effect of the surface elements and the level of detail required to determine the running surface. The selection of features is based on the findings of the measurements carried out by Mauz et al. [17] and the typical lateral position of the running surface. The following features are obtained based on the findings.

- **Average intensity** $\bar{I}$ of the reflected light within a surface element: A high intensity of reflected light corresponds to the area of the running surface.
- **Standard deviation** $\sigma_{I}$ of intensity of the reflected light within a surface element: Outside the area of the running surface, a stronger fluctuation in the detected intensity can be observed.
- **Lateral position** *y* of a surface element within the reference system of the trolley setup: The running surface is typically located on the inside of the rail and the lateral position is therefore characteristic. This is particularly relevant for rail surfaces with thin corrosion layers and potentially multiple areas of high intensity.

The machine learning models are trained with measured data from Rail 1 and tested with data from Rails 2 and 3. As a result, 33.3% of the data set is used for training and 66.7% for testing. The labeling of the data is carried out manually by three different people on the basis of the camera recordings. To avoid overfitting the lateral position of the running surface of Rail 1, the data set is augmented. The running surface is shifted 20 mm to the left and 10 mm to the right with a step width of 1 mm. In the data augmentation process, the running surface is shifted to the left by a larger amount compared to the right side as the running surface is located originally on the right-hand side of Rail 1. The procedure for determining the lateral position $y_{s}$ and the width $w_{s}$ of the running surface is as follows.

1. Measurement of the longitudinal position *x* along the rail and the lateral position *y* across the rail, as well as the distances *z* to the rail surface and the intensity of the light reflected from the surface for each measuring point.
2. The measured intensities are grouped into surface elements in the x–y plane. The surface element size is 0.5 mm × 50 mm (Δy × Δx). A longitudinal and a lateral position are assigned for each surface element.
3. An average intensity $\bar{I}$ as well as a corresponding standard deviation $\sigma_{I}$ of intensity is determined for each surface element. In combination with the lateral position of the surface element, the mean intensity and the standard deviation serve as features for the classification.
4. A pre-trained model is applied to perform the binary classification of the surface elements as “running surface” or “no running surface”.
5. The lateral positions of the edges $y_{min}$ and $y_{max}$ of the running surface in the reference system of the trolley setup are determined based on the classification of the rail surface elements. If more than 35% of the surface elements at the same lateral position are classified as “running surface”, the entire line along the rail is assigned to the running surface. The threshold value of 35% is determined empirically based on available measurements. The threshold value can be adjusted for a more conservative determination of the running surface.
6. Calculation of the width $w_{s}$ of the running surface.
7. The reference systems of the trolley setup and the rail do not coincide. The offset of the reference systems must be determined to obtain the lateral position of the running surface in the reference system of the rail. The laser profilometer detects part of the transverse profile of the rail. The reference profile of the rail is known. The lateral offset can be determined by a curve fit and, consequently, the lateral position of the running surface is known in the reference system of the rail.

## 3. Results

**Figure 6:**
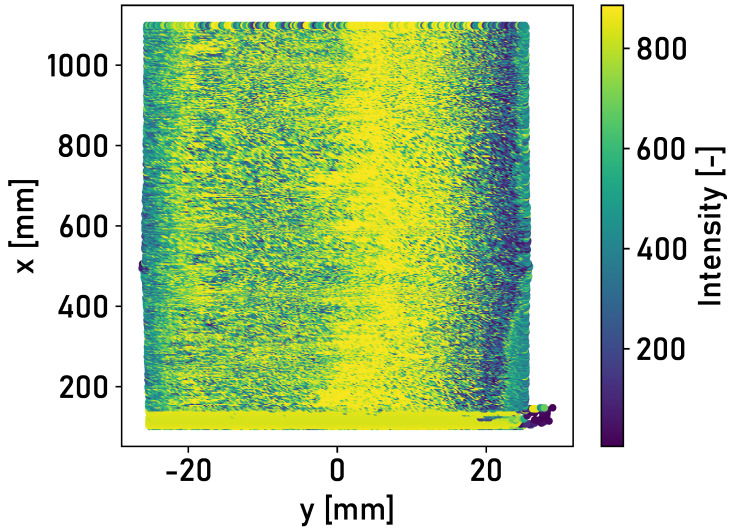
Top view of segment 0 m≤x≤1 m of Rail 1 in the central position, colored according to the detected intensities of the light reflected from the rail surface.

In laboratory tests with an artificial running surface carried out by Mauz et. al [17], a clear distinction could be made between the running surface and the corroded edge areas based on the intensity of the light reflected from the surface. The detected intensities of the three different rail surfaces from field experiments are analyzed. Figure 6 shows the detected intensities of the reflected light for the surface of Rail 1.

**Figure 7:**
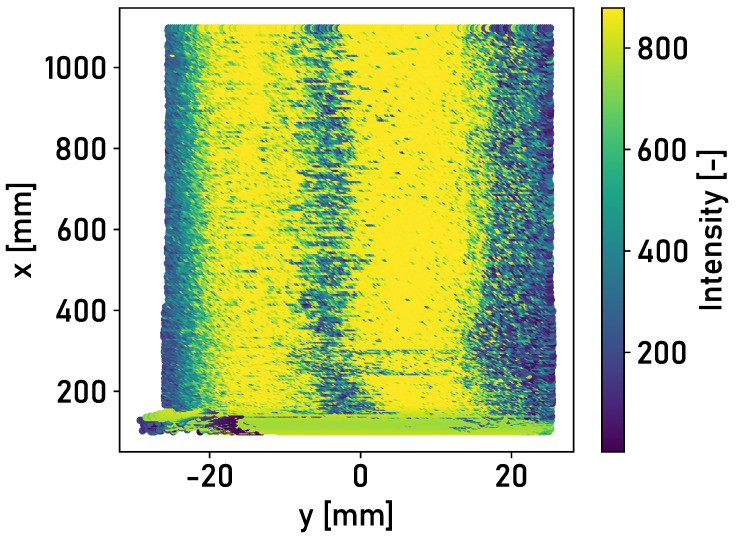
Top view of segment 0 m≤x≤1 m of Rail 2 in the central position, colored according to the detected intensities of the light reflected from the rail surface.

The area of high intensity of ≈800 corresponds to the running surface. The corroded edge areas are characterized by lower intensities of ≈400–600. This also applies to the surface of Rail 2. The trigger mark applied to the rail surface can be recognized at a longitudinal position of x≈0 m. A second area of high intensity is recognizable on the left-hand side. This is due to the thin layer of corrosion on the rail surface. Figure 7 shows the detected intensities of the reflected light for the surface of Rail 2.

**Figure 8:**
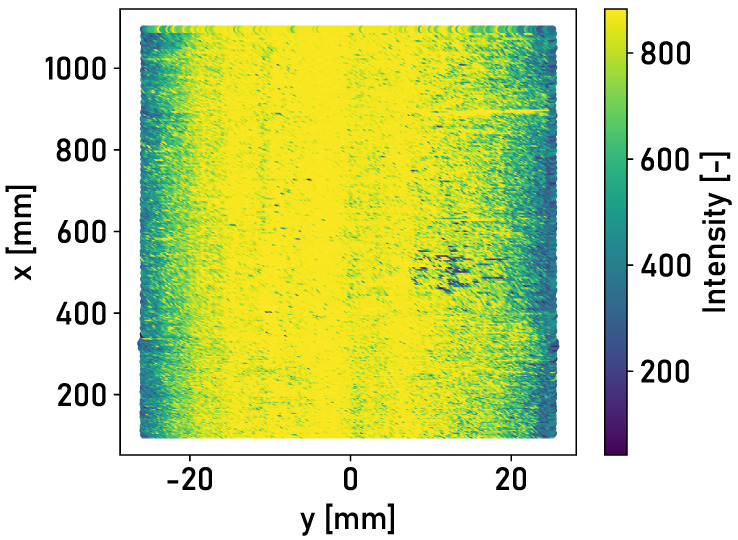
Top view of segment 0 m≤x≤1 m of Rail 3 in the central position, colored according to the detected intensities of the light reflected from the rail surface.

A large part of the surface of Rail 3 corresponds to the state of a shiny and run-in rail. It is possible to differentiate between the running surface and the corroded edge areas based on the detected intensity of the reflected light. Figure 8 shows the detected intensities of the reflected light for the surface of Rail 3.

**Figure 9:**
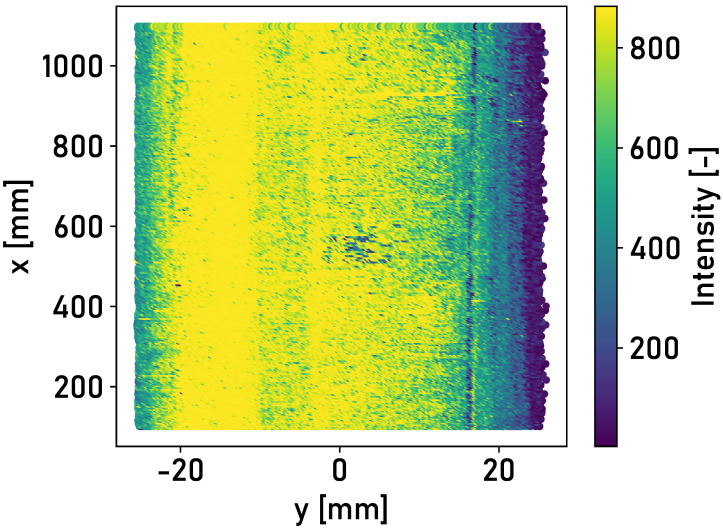
Top view of segment 0 m≤x≤1 m of Rail 3 in the right position, colored according to the detected intensities of the light reflected from the rail surface.

The distinction between different surface conditions is possible for a lateral displacement of the laser profilometer. Figure 9 shows the detected intensities of the reflected light for the surface of Rail 3 and a lateral shift of the laser profilometer to the right.

The inner edge of the rail becomes recognizable due to the lateral displacement. The detected intensity drops significantly towards the edge. This is caused by the curvature of the track surface and the associated reflection of light into the surrounding area. This can equally be observed for a shift to the left. The findings of the measurements in the laboratory environment can be confirmed.

The application of the detection methodology described in Section 2.2 is investigated in the following. The intensities of the rail surfaces in Figure 6, Figure 7 and Figure 8 are divided into surface elements. Table 1 shows the accuracy achieved for Rails 2 and 3, with different classifiers and lateral positions of the laser profilometer over the rail.

**Figure 10:**
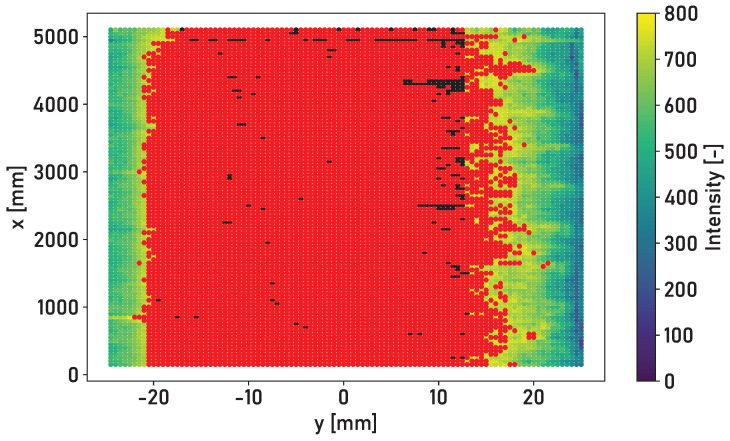
Top view of the surface of Rail 3 in the central position, with surface elements colored according to the intensity of the light reflected from the surface. Red coloring: classification of the model; black coloring: part of the running surface that was not classified as the running surface by the model.

Accuracy is defined as the ratio of the number of correctly classified surface elements to the total number of surface elements of the rail surface. The results of applying the models to Rails 2 and 3 are considered here since Rail 1 provides the training data set in a modified form. Accuracy between 76% and 86% is achieved for Rail 2 and a trained linear support vector machine. Accuracy between 90% and 94% is achieved for Rail 3 and a trained linear support vector machine. The achieved accuracy for Rail 3 is higher as it does not possess an area with a thin corrosion layer compared to Rail 2. The trained linear support vector machine is applied for the following evaluations. A mixed training data set consisting of parts of Rail 1, Rail 2 and Rail 3 does not lead to an increase in the maximum achieved accuracy. Figure 10 shows the result for the classification of the linear support vector machine for the surface of Rail 3. The red coloring of the surface elements shows those elements that are classified by the model as part of the running surface. The black coloring of the surface elements shows those elements that are manually classified as part of the running surface but not by the model.

The deviations in the determined running surface widths vary between the different lateral positions of the laser profilometer. The average deviation $\Delta w_{s}$ varies by 2.20 mm for Rail 2. The average deviation $\Delta w_{s}$ varies by 6.80 mm for Rail 3. An average width of 17 mm is determined for Rail 2 and the central position of the laser profilometer. An average width of 37.70 mm is determined for Rail 3 and the central position of the laser profilometer. The width of the running surface of Rail 3 tends to be underestimated. The deviations in the running surface width are smaller for Rail 2. This is due to the training data set, which is composed of the surface of Rail 1 and its extension via a shift in the lateral position of the running surface. The running surface of Rail 1 has a similar width to that of Rail 2. As the lateral position is included as a feature, the model has adapted accordingly. Table 2 shows the determined widths $w_{s}$ of the running surface, the lateral positions of the edges of the running surface $y_{min}$ and $y_{max}$, the manually measured widths $w_{sl}$ of the running surface and the corresponding deviations $\Delta w_{s}$.

Table 3 shows the mean values and standard deviations of the results from the manual determination of the running surface.

## 4. Discussion

The width and lateral position of the running surface are detected according to Table 2 for segments with a length of 1 m, respectively. This is relevant for an application in the field of acoustic rail roughness. The minimum evaluation length of 1 m is required by EN15610 [14], which describes the procedure for measuring the acoustic rail roughness. The length considered for the classification must be set for the respective application.

The linear support vector machine model provides the best classification results for different lateral positions and rail surfaces. The maximum accuracy of 94% achieved for Rail 3 is sufficient to determine the relevant parameters of the width and lateral position of the running surface. The decisive factor is the formation of clusters on the surface, which makes it possible to differentiate between the surface conditions. Ideal accuracy of 100% is not required for this purpose. The distinction of the edge area can be influenced by the choice of the threshold value for the proportion of the surface elements that must be classified as “running surface” at an identical lateral position. The more conservative determination of the running surface is feasible to reduce the relative deviations in the determined width of the running surface shown in Table 2. Due to the less sharp distinction of the corroded edge area for Rail 3, a larger relative deviation in the determined width of the running surface between 15.15% and 19.05% is calculated. A relative deviation between −2.94% and 12.12% is determined for Rail 2.

While accuracy between 76% and 86% is achieved when applying the linear support vector machine for Rail 2, the accuracy for Rail 3 increases to a range between 90% and 94%. The lower accuracy is due to the different surface conditions of Rails 1 and 2. The detected intensity determines two areas of high intensity for Rail 2, as shown in Figure 7. The thin corrosion layer appears as a second running surface in the measured intensity. The training data set applied is the surface of Rail 1, which contains one area of higher intensity. The training data should be extended in the future to include various rail surface conditions and forms of running surfaces.

**Figure 11:**
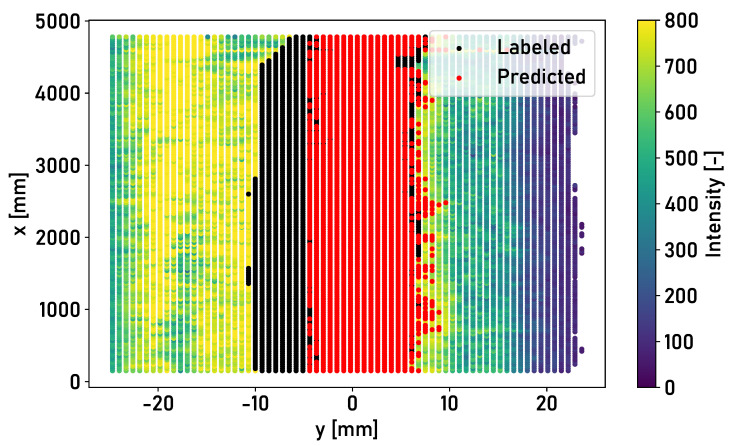
Top view of the surface of Rail 3 in the central position, with surface elements colored according to the intensity of the light reflected from the surface. Red coloring: classification of the model trained by data of Rail 1; black coloring: part of the running surface that was not classified as the running surface by the model.

The shape of the running surface in Rail 1, Rail 2 and Rail 3 differs both in width and in the quality of the surface condition. Overfitting of the lateral position of the surface elements occurs if only Rail 1 is chosen for the training of the model. Figure 11 shows the classification result for the surface of Rail 2 for a model that is trained without data augmentation, exclusively with measurement data from Rail 1.

The running surface detected by the model is laterally limited. From a lateral position <−5 mm, no more surface elements are assigned to the running surface. The inclusion of the lateral position of the surface elements should be retained as a feature, as the running surface is typically located towards the inner edge of the rail. Overfitting is prevented in the presented investigations by data augmentation, which leads to a detection rate of >87% for Rail 3. For network-wide application, the training data set must be extended by measurement data from the rail network, which include different lateral positions of the running surface.

The difference between the reference system of the measurement setup and the rail is determined by a curve fit of the measured cross-profile with the reference profile of the rail. In the future, this can be achieved by the selection of a laser profilometer with a longer projected line transverse to the direction of travel.

The presented methodology can be applied to an onboard installation on a train. The investigation of external disturbances is essential for the application of the running surface detection methodology as part of an onboard monitoring system. This includes vertical movements such as shocks or vibrations of the vehicle, which can be emulated on a test stand, as well as moisture on the rail surface. These external interferences can possibly lead to the distortion of the measurement result and must be considered in isolation under laboratory conditions, and compensation techniques must be developed. Immediately after grinding the rail surface, no clear running surface is formed. Consequently, the optical differences between the corroded and non-corroded surface are not visible to the laser profilometer. In this case, the detection of the area of the rail surface on which the wheel has run is only possible to a limited extent.

## 5. Conclusions

It is shown that the detection of the running surface can be achieved with a laser profilometer based on the intensity of the light reflected from the rail surface. The results from the laboratory environment are verified for various rail surface conditions with different levels of wear. The application of supervised machine learning models enables the automatic detection of the lateral position and the width of the running surface. The mean intensity, the standard deviation of the intensity and the lateral position of a surface element on the rail surface are applied as features. A surface element size of 0.5 mm × 50 mm (Δy × Δx) is set. The classifier applied is a linear support vector machine that achieves accuracy of over 90% for a shiny rail surface. The average deviation in the determined width of the running surface of a retracted shiny rail surface varies between −1.2 mm and 5.6 mm for different lateral positions of the sensor. The detection methodology offers the possibility for the network-wide detection of the running surface for the condition monitoring of the rail network. This can be realized by the onboard installation of the setup and can furthermore enable measurements at the speed of the rail vehicle. This would provide the basis for the implementation of a direct optical measurement setup for rail roughness measurements on the rail vehicle. For this purpose, the robustness of the detection methodology against external disturbances such as vertical vehicle movements must be investigated. For application in the condition monitoring of the rail network, the extension of the training data set is necessary, which would enable the implementation of further machine learning models, e.g., the application of deep learning.

## Figures and Tables

**Table 1:** The accuracy achieved for the random forest, k-neighbors and linear support vector machine models, with different lateral positions of the laser profilometer and for the rail surfaces of Rails 2 and 3.

		Random Forest [%]	K-Neighbors [%]	Linear Support Vector Machine [%]
	Left	90.00	88.00	86.00
Rail 2	Central	74.00	76.00	79.00
	Right	77.00	77.00	76.00
	Left	88.00	89.00	94.00
Rail 3	Central	87.00	87.00	90.00
	Right	87.00	87.00	92.00

**Table 2:** Determined lateral positions of the running surface at the central position of the laser profilometer. $y_{min}$: lateral position of the left edge of the running surface; $y_{max}$: lateral position of the right edge of the running surface; $w_{s}$: width of the running surface; $w_{sl}$: manually determined width of the running surface; $\Delta w_{s}$: deviation between the manual and automatic determination of the width of the running surface.

	Longitudinal	$y_{\min}$	$y_{\max}$	$w_{s}$	$w_{\mathrm{sl}}$	$\Delta w_{s}$	Relative Error
	Position	[mm]	[mm]	[mm]	[mm]	[mm]	[mm]
Rail 2	0 m≤x≤1 m	−0.15	16.85	17	16.5	0.5	3.03%
	1 m≤x≤2 m	−2.45	16.05	18.5	16.5	2	12.12%
	2 m≤x≤3 m	0.26	16.76	16.5	16	0.5	3.13%
	3 m≤x≤4 m	−0.15	16.35	16.5	16.5	0	0.00%
	4 m≤x≤5 m	1.35	17.85	16.5	17	−0.5	−2.94%
Rail 3	0 m≤x≤1 m	−20.50	17.50	38	33	5	15.15%
	1 m≤x≤2 m	−20.50	17.50	38	32.5	5.5	16.92%
	2 m≤x≤3 m	−20.50	18.00	38.5	32.5	6	18.46%
	3 m≤x≤4 m	−20.50	17.00	37.5	31.5	6	19.05%
	4 m≤x≤5 m	−19.50	17.00	36.5	31	5.5	17.74%

**Table 3:** Summary of the mean deviations of the running surface widths from the manual determination and the associated mean relative errors. $\Delta w_{s}$: deviation between the manual and automatic determination of the width of the running surface.

		Rail 2		Rail 3	
		Average	St. Dev.	Average	St. Dev.
Left	$\Delta w_{s}\left[mm\right]$	2.60	1.11	2.60	1.36
	Relative Error [%]	16.16	6.78	9.06	4.73
Central	$\Delta w_{s}\left[mm\right]$	0.50	0.84	5.60	0.37
	Relative Error [%]	3.07	5.05	17.47	1.36
Right	$\Delta w_{s}\left[mm\right]$	0.40	1.74	−1.20	4.95
	Relative Error [%]	1.98	12.38	−3.01	12.54

## Data Availability

The data used in this study are available from the corresponding author upon request.
